# Retraction: Inhibitory potentials of *Streptomyces exfoliatus* strain ‘MUJA10’ against bacterial pathogens isolated from rural areas in Riyadh, Saudi Arabia

**DOI:** 10.1371/journal.pone.0298008

**Published:** 2024-01-25

**Authors:** The PLOS ONE Editors

The *PLOS ONE* Editors retract this article [1] because it was identified as one of a series of submissions for which we have data concerns and concerns about authorship, peer review, and data availability. We regret that the issues were not addressed prior to the article’s publication.

The author either did not respond directly or could not be reached.
